# Antimicrobial titanium/silver PVD coatings on titanium

**DOI:** 10.1186/1475-925X-5-22

**Published:** 2006-03-24

**Authors:** Andrea Ewald, Susanne K Glückermann, Roger Thull, Uwe Gbureck

**Affiliations:** 1 Department for Functional Materials in Medicine and Dentistry, University of Würzburg, Pleicherwall 2, D-97070 Würzburg, Germany

## Abstract

**Background:**

Biofilm formation and deep infection of endoprostheses is a recurrent complication in implant surgery. Post-operative infections may be overcome by adjusting antimicrobial properties of the implant surface prior to implantation. In this work we described the development of an antimicrobial titanium/silver hard coating via the physical vapor deposition (PVD) process.

**Methods:**

Coatings with a thickness of approximately 2 μm were deposited on titanium surfaces by simultaneous vaporisation of both metals in an inert argon atmosphere with a silver content of approximately 0.7 – 9% as indicated by energy dispersive X-ray analysis. On these surfaces microorganisms and eukaryotic culture cells were grown.

**Results:**

The coatings released sufficient silver ions (0.5–2.3 ppb) when immersed in PBS and showed significant antimicrobial potency against *Staphylococcus epidermis *and *Klebsiella pneumoniae *strains. At the same time, no cytotoxic effects of the coatings on osteoblast and epithelial cells were found.

**Conclusion:**

Due to similar mechanical performance when compared to pure titanium, the TiAg coatings should be suitable to provide antimicrobial activity on load-bearing implant surfaces.

## Background

Deep infection of endoprostheses remains a serious complication in orthopedic surgery since the systemic treatment of infected bone with antibiotics is often impossible due to the poor accessibility of the infection site by applied antibiotics [1]. The primary infection rate in total hip arthroplasty was found to be approximately 1–3% [2] and the rate of re-infection after revision of infected hip prostheses is up to 14% [3]. The reason for a post-operative infection is mainly due to a contamination of the implant surface during implantation with the formation of a resistant biofilm as well as a haematogenic bacterial spreading (e.g. after tooth extraction) [4]. Chronically infected endoprostheses have to be explanted, common therapies involve the use of temporary antibiotic loaded PMMA spacer-implants [5,6] or antibiotic loaded cements [7]. Although these therapies show good results and reimplantation is successful in most cases, a risk of creating bacterial resistance due to low releasing doses persists [5-7].

Post-operative infections may be overcome by adjusting antimicrobial properties of the implant surface prior to implantation. Techniques described in literature are direct impregnation with antibiotics [8] prior to implantation or using antibiotic or silver doped polymer coatings. Silver-based antimicrobials are of interest due to the non-toxicity of the active Ag^+ ^to human cells [9,10] and the antimicrobial activity of silver ions has been well established. Silver ions are significant antimicrobials with only few bacteria being intrinsically resistant to this metal through plasmid derived resistance mechanisms [11-14]. Incorporation of silver ions into polymeric materials has been widely used for several years; especially urinary and central venous catheters are provided with silver coatings to reduce infections. Medical devices like heart valves or dialysis units also benefit from the use of silver doted surfaces [15-18].

However, most of the techniques used do not fulfil the mechanical criteria for load-bearing implants because especially the implantation into the bone results in high (abrasive) shear forces between bone and the implant surface. The aim of this study was the development of a metallic hard coating based on titanium/silver alloys which provides antimicrobial properties due to the silver content as well as a high biocompatibility in contact with bone. Titanium samples were coated with the silver-titanium-alloys containing up to 9% silver by using the physical vapour deposition (PVD) process and tested for hardness, biocompatibility and bactericide action. The PVD process is known to result in good adhesiveness and wear resistance of metallic or ceramic coatings and is widely used in technical and medical applications [19]. The coatings are aimed to provide antimicrobial properties due to the release of silver ions in an aqueous environment while maintaining the biocompatibility and hardness of titanium against hard and soft tissue for an application on load-bearing implants, e.g. in hip or knee arthroplasty.

## Materials and methods

### Sample preparation

Cylindrical titanium specimens (16 mm diameter × 1 mm height) were cut from technical grade titanium sheet (ASTM-Nr. B 265–95, Grade 2, Zapp, Düsseldorf, Germany). The specimens were cleaned ultrasonically in pure water followed by alkaline Extran^®^-solution and an additional incubation in pure water for 10 min at 40°C each. Drying and storage of the specimens occurred under clean room conditions until positioning in the recipient. The titanium/silver coatings were manufactured by using a PVD-system type PLS 570 (Pfeiffer Vacuum, Germany). Vaporisation of the metal components occurred by means of an arc (titanium) and a magnetron sputter (silver) from metallic targets. Mixed coatings were produced by simultaneous vaporisation of both metals while the titanium substrate was rotated with a frequency of 0.5 Hz. After evaporation the specimens were physically cleaned in an argon plasma (p = 6.4·10^-3 ^mbar; 200 W) for 30 min and through this heated to the start temperature of approximately 200°C. Processing parameters for all coatings were an argon atmosphere of 7.5·10^-3 ^mbar, a substrate voltage U_S _of -300 VDC, a coating time of 30 min and a constant arc current (for vaporising the titanium component) of 80A while the magnetron powder (silver component) was varied between 112 – 900 W to modify the ratio between the two vaporized metal components.

### Surface characterisation

The surface roughness was measured with a profilometer *Surftest 211 *(Mitutoyo, Japan); 3 measurements were carried out at various positions on the surface, the average and the standard deviation were calculated. The hardness of the coatings was determined by measuring the Vicker's hardness HV10 (Zwick, Ulm, Germany). SEM-analyses were performed with a DSM 940 (Zeiss, Oberkochen, Germany). EDX-analyses of the surfaces were recorded with a QX2000 system (Link, England) at a working distance of 25 mm and an acceleration voltage of 10 kV. The EDX spectra were quantified using the internal ZAF software. The silver ion release of the coatings was measured with inductively coupled plasma mass spectroscopy (ICP-MS). Samples (n = 3 per condition) were immersed in 10 ml phosphate buffered saline (PBS, 137 mM NaCl, 2.7 mM KCl, 7.0 mM Na_2_HPO_4 _× 2 H_2_O, 1.5 mM KH_2_PO_4_) at 37°C. The buffer solution was replaced and analysed after 1 d, 2 d, 4 d, 10 d and 14 d with a Varian ICP-MS against standard solutions (Merck, Darmstadt, Germany).

### Antimicrobial properties

For analysing the antimicrobial properties of the titanium/silver coatings samples were exposed to gram positive *Staphylococcus epidermidis*, strain RP62A (ATCC 35984) or gram negative *Klebsiella pneumoniae*, strain 3091 [20]. The microorganisms were grown in LB broth (1% w/v Tryptone, 0.5% w/v Yeast extract, 0.5% w/v NaCl) at 37°C. Titanium samples were placed into 24 well plates (Nunc, Wiesbaden, Germany), covered with 1 ml bacteria suspension in logarithmic growth phase and incubated by gentle rotation for 24 hours at 37°C. Adhered organisms were dehydrated in 50%, 80%, and two times 90% Ethanol for 5 min each. The specimens were air dried and incubated for 15 min with 1 ml SYBR^®^-green (Molecular probes™, Invitrogen Life Technologies, Karlsruhe, Germany) diluted 1:100 000 in TBS (10 mM Tris-HCl, pH 7.4; 140 mM NaCl). Adhered microorganisms were quantified by evaluating the fluorescence in a Spectrafluor plus photometer (Tecan, Crailsheim, Germany) with the appropriate calculation software (X-Fluor, Tecan, Germany). Three samples were analyzed per surface modification and average and standard deviation were calculated. Statistical analysis was performed by using the Anova t-test of Microsoft Excel.

### Cell culture

Epithelial cells 16HBE were cultured in Dulbecco's Modified Eagle's Medium (DMEM, Invitrogen Life Technologies, Karlsruhe, Germany), human osteoblast cell line hFOB1.19 [21] was cultured in DMEM/NUT MIX F-12 (HAM, Invitrogen Life Technologies). The culture media were supplemented with 10% fetal calf serum, 1% penicillin and streptomycin, respectively (Invitrogen Life Technologies) and the cells incubated in a humidified 5% CO_2 _incubator at 37°C. For biocompatibility testing the samples were placed in triplicate into a 24-well plate (Nunc, Wiesbaden, Germany), cells were seeded onto silver containing titanium surfaces or on polystyrene, respectively, with an initial density of 50 000 cells per well.

### Biocompatibility testing

For testing the biocompatibility of titanium/silver surfaces cell proliferation, cell viability and total protein content of the samples were determined after 3, 5, 7, and 10 days of culture. Cell proliferation was determined by electronic cell counting using a CASY 1 TTC cell analyzer (Schärfe System, Reutlingen, Germany). For this purpose, cells were detached by incubation with Accutase (PAA, Cölbe, Germany) after two washes in PBS. The reaction was stopped by adding equal amounts of DMEM. After diluting the cell suspension 1:100 in 10 ml Isoton III (Beckmann Coulter, Krefeld) cells were counted and the number was calculated automatically by the Casy-stat software (Schärfe System). Cell viability and proliferation was analysed by using cell proliferation reagent WST 1 (Roche Diagnostics, Mannheim, Germany). After incubating the cells for 30 min with the WST reagent 1:10 in DMEM at 37°C, the adsorption of the supernatant was quantified in a Tecan spectra fluor plus photometer (Tecan, Crailsheim, Germany). Total protein content of the cells was quantified applying the DC Protein Assay (BioRad, Munich, Germany) according to manufacturers' instructions. The adsorption was measured in a Tecan spectra fluor plus photometer. For each analysis the samples were examined in triplicate and the average and standard deviation was calculated. For statistical analysis the Anova t-test of Excel was performed.

## Results

**Figure 1:**
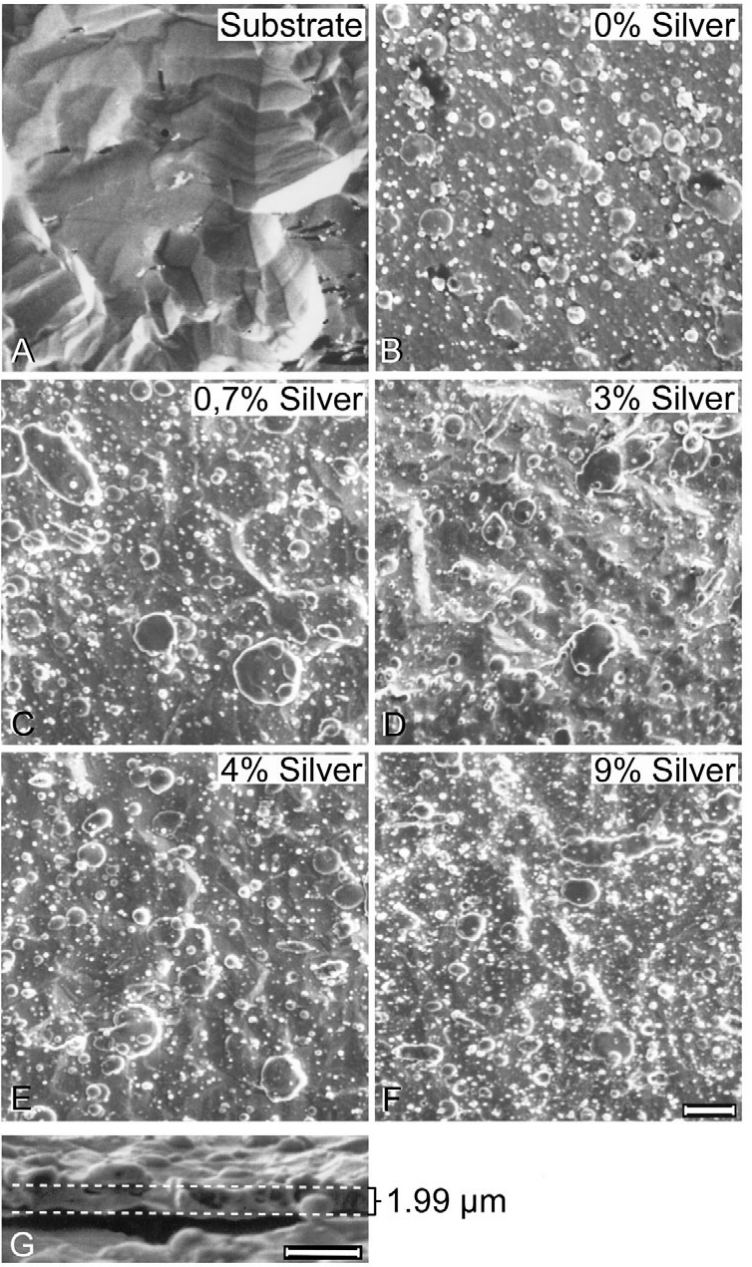
**EMOF**. Scanning electron micrographs of a titanium surface (A) and titanium surfaces coated with TiAg alloys. B 0% Ag, C 0.7% Ag, D 3% Ag, E 4% Ag and F 9% Ag. (bar = 10 μm for A – F). The thickness of the coating is about 2 μm as determined by electron microscopy (G, bar = 5 μm).

**Figure 2:**
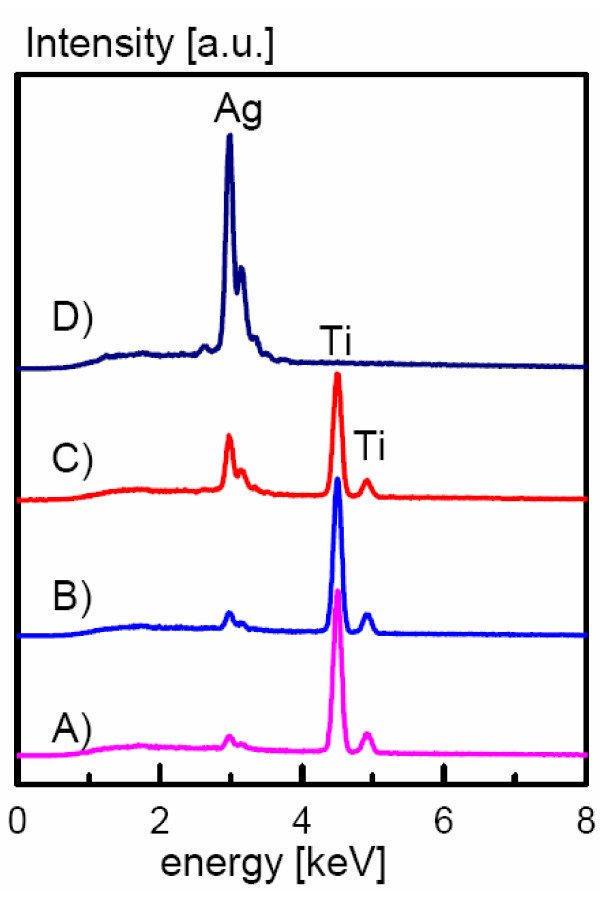
**EDX**. EDX-analysis of TiAg-PVD coatings on titanium; A) 3%, B) 4%, C) 9% and D) 100% (silver contents were were obtained by quantifying the EDX spectra using the ZAF software).

**Figure 3:**
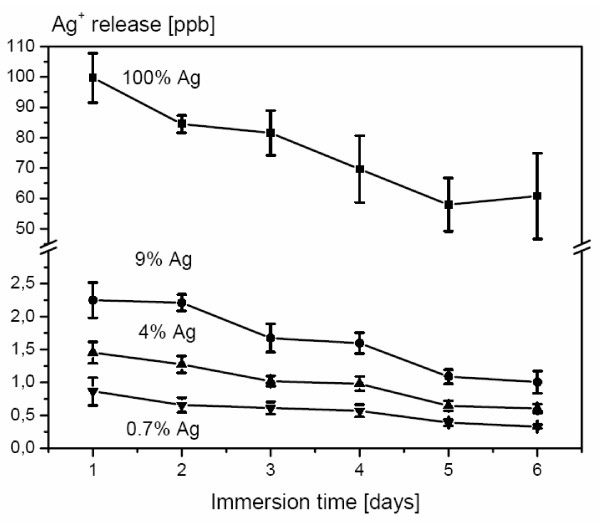
**ICP**. Ag^+ ^release from TiAg coatings over 6 days (n = 3); the immersion liquid (PBS) was replaced daily.

PVD coating of titanium resulted in the deposition of thin metallic films with a thickness of approximately 2 μm as determined from cross-section images. SEMs of the coatings are displayed in Figure 1. Independent of silver content, the surfaces showed similar surface structures. EDX spectra of the coatings (Figure 2) showed the presence of small amounts of silver within the surface. Quantification of the spectra using the ZAF software indicated a silver content between 0.7% and 9%. The appropriate composition was found to be homogeneous all over the surface as screened by EDX analysis (with a lateral resolution of approximately 100 μm^2^) at different surface positions. The coating procedure increased the medium surface roughness R_y _only marginally (Table 1) from 2.8 μm to 4.5 μm (9% silver content) and the coatings provided Vickers hardness in the same range as unmodified titanium. When immersed in PBS, the surfaces continuously released silver ions (Figure 3); concentrations determined by ICP-MS analysis were in the range between 0.5 to 2.3 ppb at 0.7 – 9% silver content compared to 60–100 ppb for a pure silver reference surface.

**Table 1:** Surface roughness and Vickers hardness HV10 of TiAg-coated titanium surfaces (n = 3).

Ag content [%]	R_y _[μm]	HV10
titanium substrate	2.80 +/- 0.21	193 +/- 1.67
0.7	3.79 +/- 0.62	215 +/- 4.18
3	4.16 +/- 0.23	189 +/- 3.21
4	4.13 +/- 0.27	205 +/- 7.60
9	4.56 +/- 0.80	214 +/- 3.63
100	2.98 +/- 0.12	188 +/- 3.96

### Antimicrobial properties

**Figure 4:**
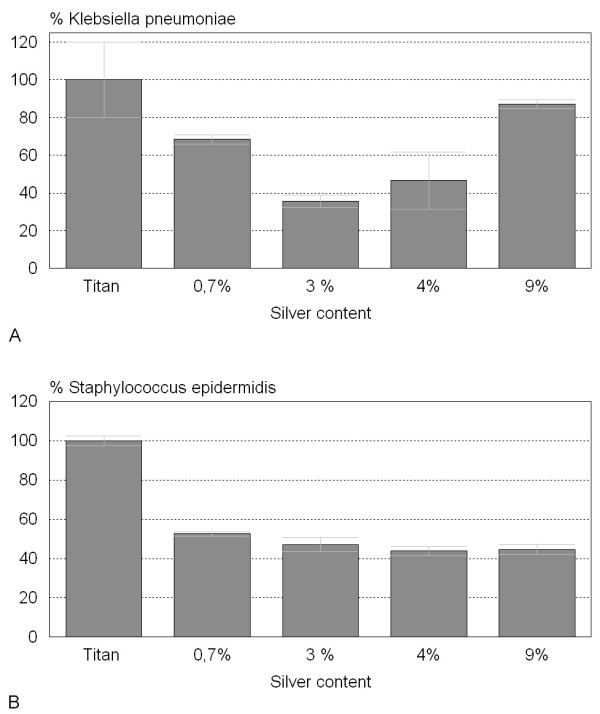
**Bact**. Adhesion of bacterial strains on argentiferous surfaces. A) Klebsiella pneumoniae adhesion is significantly (p < 0.05%) reduced on surfaces containing 3% silver. B) Staphylococcus epidermidis adhesion is significantly (p < 0.01%) reduced on all of the silver containing surfaces.

Figure 4 shows the reaction of *Staphylococcus epidermis *and *Klebsiella pneumoniae *strains to silver doted surfaces compared to commercial pure titanium grade 2 as control. Bacterial adherence to control surfaces was set to 100% and the contamination on the argentiferous surfaces was calculated accordingly. *Klebsiella pneumoniae *showed a significant reduction of adhesion (p < 0.05) on 0.7% up to 4% silver surfaces ranging from 32% to 64%. The decrease of microorganism adhesion on 9% silver was not significant for *Klebsiella*. The dwindling of *Staphylococcus epidermidis *adhesion was significant (p < 0.01) on all surfaces tested ranging from 52% on 0.7% silver to 43% on 4% silver.

### Biocompatibility properties

**Figure 5:**
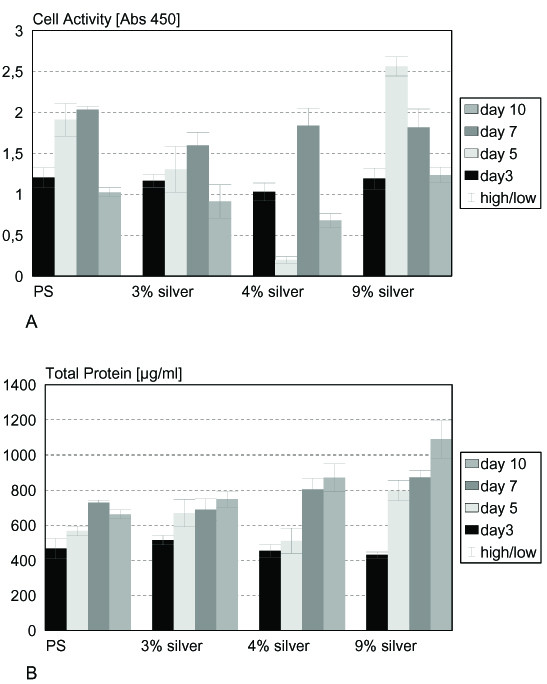
**Epi**. Cytotoxicity tests with epithelial cells. A) Epithelial cell activity is not significantly (p < 0.01%) reduced on silver containing surfaces. B) Epithelial cell protein content is not significantly (p < 0.01%) reduced on silver containing surfaces.

**Figure 6:**
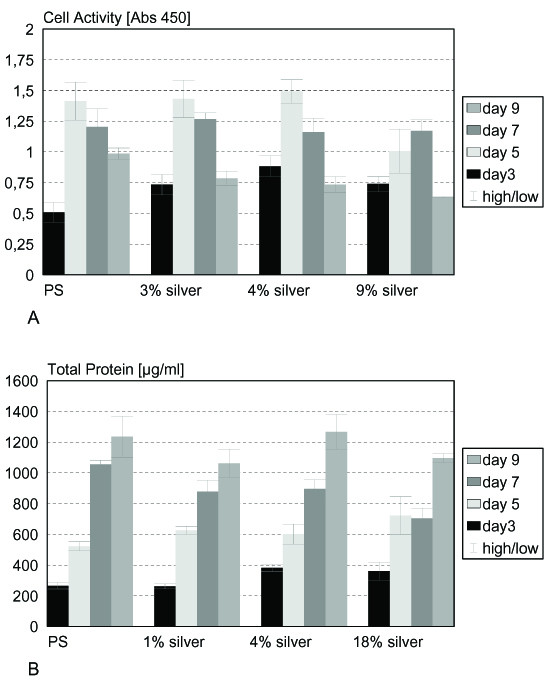
**Osteo**. Cytotoxicity tests with osteoblast cells. A) Osteoblast cell activity is not significantly (p < 0.01%) reduced on silver containing surfaces. B) Osteoblast protein content is not significantly (p < 0.01%) reduced on silver containing surfaces.

Biocompatibility tests were performed using epithelial and osteoblast cell lines, both predestinated for getting in contact with implant surfaces. Especially osteoblast cells are expected to grow directly on the implant surface in contact with bone. Epithelial cell activity showed a significant reduction on 3% silver surfaces after 5 and 7 days and on 4% silver surfaces after 5 and 10 days, on 9% silver the cell activity was increased significantly on day 5 and 10 (p < 0.01) (fig. 5A). Determination of total cell protein on the various surfaces yielded a significant increase of protein on 3% and 9% silver containing surfaces after 5 and 10 days as well as on 4% silver surfaces on day 7 and 10 and on 9% silver after 7 days (p < 0.01) (fig. 5B). Figure 6 summarizes the results for the osteoblast cell line. Cells showed comparable or significantly higher activity on most of the surfaces tested (3% silver day 3, 4% silver day 3, 9% silver day 3; p < 0.01). Cell activity is significantly decreased (p < 0.01) on 3% silver after 10 days and on 9% silver after 5 and 9 days (fig. 6A). Total protein content of osteoblasts cultivated on argentiferous titanium was increased on 1% silver on day 5, on 4% silver on day 3 and 5, and on 18% silver on day 3 (p < 0.01). On 1% silver (day 7 and 9), 4% (day 7), and 18% (day 7 and 9), total protein content was decreased (p < 0.01) when compared to pure titanium surfaces (fig. 6B). According to DIN EN ISO 10993 and to ISO 7405:1997, the silver doted titanium surfaces showed no cytotoxicity on epithelial and osteoblast cells.

## Discussion

Biofilm formation is a recurrent complication in implant surgery. Implanted devices are ideal substrates for biofilm forming microorganisms, which colonise short term implants such as catheters as well as long term prostheses like artificial hip or knee joints [22]. The biofilm formation proceeds successively. In a first step, microorganisms associate transiently to the surface, followed by a robust adhesion. After aggregation and formation of microcolonies the biofilm grows and matures. During this process proteins, glycoproteins and, carbohydrates are incorporated and microorganisms cease division [23,24]. As antibiotics perform their bactericidal features by impairing cell division processes, non-dividing microorganisms living in biofilms are no longer susceptible to antibiotics and exhibit a transient resistance to these substances. On the other hand the compact structure of biofilms constitutes a diffusion barrier for antibiotics [25-27].

Since it has been shown that microorganisms and host cells compete for the substrate in a process called "race for the surface" [28], device surfaces should prevent microorganisms from adhering in order to promote host tissue interaction with the implant. Antimicrobial properties of implant surfaces can be obtained by either impregnation of the surface with antibiotics [29] or modification of the surface or the bulk material with silver which is slowly released during the application [30,31]. For this reason we hypothesised that coating of titanium with a silver/titanium alloy will result in bacteria repellent properties while maintaining the biocompatibility to eukaryotic cells. The low resistance rate in bacteria toward silver ions is advantageous compared to antibiotics modified surfaces. Although plasmid derived resistance in microorganisms living in halide containing environments has been described, up to now only few cases of silver resistant bacteria were found in hospital [14]. The antimicrobial activity of silver is dependent on the silver cation Ag^+^, which strongly binds to electron donor groups in biological molecules containing sulphur, oxygen or nitrogen. The biological performance of silver ions is dose dependent. Low concentrations <35 ppb are mainly bactericide, while higher doses can be a toxic to human cells, ranging from low or high local toxicity to systemic effects like argyria in high doses (>4–6 g total silver content in the body) [40]. Hence the silver-based antimicrobial materials have to release Ag^+ ^ions to create a pathogenic environment in order to be effective. The oxidation of metallic silver to the active species (Ag^+^) is mediated through an interaction of the silver with the aqueous environment. Silver coatings are commonly applied on polymer-implants and recently, the silver coating of foreign materials such as heart valves, central venous catheters and urinary catheters has proved to reduce the infection rate of medical devices [15-18]. Currently, no titanium based material utilizing the antimicrobial qualities of silver ions is available for load bearing implants, like artificial joint replacements.

In this work, we developed a coating method for metallic surfaces with titanium/silver modifications by using the physical vapor deposition (PVD) technique. During this procedure, a defined mixture of silver and titanium is deposited onto the surface by simultaneous vaporization of metallic targets in vacuum. The resulting samples displayed surface roughness and hardness comparable to pure titanium, a silver content of approximately 0.7 – 9% within the coatings was confirmed by EDX analysis. According to the TiAg phase diagram [32], it is likely that at low silver concentrations < 3% a solid solution of Ag in α Ti was formed during coating deposition, while at higher concentrations a binary phase mixture of α Ti and Ti_2_Ag should be present. A constant and prolonged release of silver ions at concentration levels of at least 0.1 ppb [33] is the minimum requirement for a bacteria repellent surface. In this study Ag^+ ^ions were formed by oxidation of the surface in contact with an aqueous electrolyte. Mechanistically, the silver/titanium alloy forms a galvanic element in which silver is the anode and titanium the cathode and therefore titanium is oxidised as less noble element. However, the titanium is rapidly passivated during this process with a thin TiO_2 _coating which is non-conductive and inhibits further electron transfer into the electrolyte. From this time point on, the silver is simply chemically oxidised and silver ions are released into the electrolyte. Even at the lowest silver content (0.7%) Ag^+ ^concentrations released into the medium are more than 5 fold higher than the concentrations necessary for antimicrobial activity as determined by ICP mass spectrometry. Since Ag^+ ^is able to form low soluble salts with chloride or sulfide ions, this might reduce the Ag^+ ^concentration in the electrolyte and hence reduce antimicrobial activity. Assuming a Cl^- ^concentration of approximately 160 mmol in PBS or serum and a solubility product of AgCl of 10^-10 ^mol^2^/l^2^, the concentration of free silver ions in serum is estimated to be 0.27 ppb under equilibrium conditions, which is still above the minimum concentration necessary for bacterial inhibition. Since the surfaces are intended to exhibit microorganism repellent properties only locally in order to prevent biofilm formation on the implant, binding of Ag^+ ^to serum proteins should be irrelevant.

To examine the antimicrobial properties of the argentiferous surfaces, two bacterial strains, frequently involved in nosocomial infections, were chosen. *Staphylococcus epidermidis *is a commensalistic skin microorganism which is not pathogenic in healthy individuals, but can cause serious wound and implant infections after implant surgery [34-36]. *Klebsiella pneumoniae *is also known to be involved in implant associated infections [18,37,38]. On 3% silver the colonization of the surfaces was reduced by 64% for *Klebsiella pneumoniae *surfaces. The adhesion of *Staphylococcus epidermidis *was reduced by about 50% on all surface concentrations. Surprisingly, no obvious dependency of bacterial contamination decrease on the released silver ion concentration was found. This could be due to the reaction of the silver ions with halogenide ions in the serum resulting in the formation of unreactive AgCl precipitate, which is thought to reduce the concentration of free silver ions for all surfaces to approximately 0.27 ppb as calculated in the previous paragraph. The bacterial strains used in our test system showed different sensitivities to silver ions. Mechanistically, the mode of action at low silver concentrations is an interference of Ag^+ ^with the murein of the bacterial cell wall [39]. Differences in sensitivity are therefore very likely dependent on the type of bacteria since *Staphylococcus epidermidis *is a gram positive organism whereas *Klebsiella pneumoniae *is gram negative.

At the same time the surfaces showed no cytotoxicity for eukaryotic cells like epithelial or osteoblast cells. The cytotoxicity for eukaryotic cells was tested according to DIN EN ISO 10993-5 by measuring cell activity and total protein content of the cells. Reference surfaces were polystyrene from the tissue culture dish. Epithelial cells showed comparable activity on all surfaces tested. Only the sample grown on 4% silver had a drastically reduced activity after five days. Since the cells grew properly on day 9, this phenomenon may be due to an early detachment of cells. The assay for total protein content showed reasonable results and the protein level was not reduced on any surface. Cell activity tests for osteoblasts yielded equal results on polystyrene, 3%, and 5% silver surfaces, respectively. Only on 9% silver surfaces the cell activity was reduced on day 5. The total protein content of osteoblasts grown on argentiferous surfaces is comparable among the different samples, showing no cytotoxic effects of the surfaces on eukaryotic cells. This is in agreement with a recent in vivo study, where silver coated titanium implants (Mutars^® ^prostheses) have been placed into rabbits. Compared to titanium prostheses, the silver coated prostheses showed significantly lower infection rates with *Stapylococcus aureus*, without showing toxic side effects on the surrounding tissues [40]. Similar results were obtained in other studies with silver coated polymer surfaces and stainless steel pins also showing no cytotoxicity [31,41].

## Conclusion

In this work we managed to produce a hard coating suitable to provide metal surfaces with antibacterial properties. Titanium/silver hard coatings were established via physical vapor deposition with significant antimicrobial potency and the absence of cytotoxical effects on osteoblasts and epithelial cells. In future this surface modification may be applied to load bearing implants. Further work should be focused on an increase of the hardness of the coatings, e.g. by sputtering in reactive atmosphere (oxygen, nitrogen) which would lead to TiAg(O,N) coatings similar to TiZr or TiNb coatings as described previously by our group [19]. A further simplification of the coating process may be achieved by using single target made from a titanium/silver alloy.

## Competing interests

The author(s) declare that they have no competing interests.

## Authors' contributions

AE took part in conceiving of the study, carried out the bacterial studies and drafted parts of the manuscript. SKG carried out the cell culture experiments including statistical analysis. RT participated in the design of the study and helped to draft the manuscript. UG took part in conceiving of the study, performed the surface analysis studies and drafted parts of the manuscript.
